# Using temperature coefficients to support resonance assignment of intrinsically disordered proteins

**DOI:** 10.1007/s10858-024-00452-9

**Published:** 2024-12-07

**Authors:** Paulina Putko, Javier Agustin Romero, Christian F. Pantoja, Markus Zweckstetter, Krzysztof Kazimierczuk, Anna Zawadzka-Kazimierczuk

**Affiliations:** 1 Centre of New Technologies, University of Warsaw, Banacha 2C, 02-097 Warsaw, Poland; 2 Biological and Chemical Research Centre, Faculty of Chemistry, University of Warsaw, Żwirki i Wigury 101, 02-089 Warsaw, Poland; 3 German Center for Neurodegenerative Diseases (DZNE), 37075 Göttingen, Germany; 4 Department for NMR-based Structural Biology, Max Planck Institute for Multidisciplinary Sciences, 37077 Göttingen, Germany

**Keywords:** Temperature coefficients, Intrinsically disordered proteins, Tau protein

## Abstract

The resonance assignment of large intrinsically disordered proteins (IDPs) is difficult due to the low dispersion of chemical shifts (CSs). Luckily, CSs are often specific for certain residue types, which makes the task easier. Our recent work showed that the CS-based spin-system classification can be improved by applying a linear discriminant analysis (LDA). In this paper, we extend a set of classification parameters by adding temperature coefficients (TCs), i.e., rates of change of chemical shifts with temperature. As demonstrated previously by other groups, the TCs in IDPs depend on a residue type, although the relation is often too complex to be predicted theoretically. Thus, we propose an approach based on experimental data; CSs and TCs values of residues assigned using conventional methods serve as a training set for LDA, which then classifies the remaining resonances. The method is demonstrated on a large fragment (1-239) of highly disordered protein Tau. We noticed that adding TCs to sets of chemical shifts significantly improves the recognition efficiency. For example, it allows distinguishing between lysine and glutamic acid, as well as valine and isoleucine residues based on ${{\text {H}}^{\text {N}}}$, N, ${{\text {C}}_\alpha }$ and C$^{\prime }$ data. Moreover, adding TCs to CSs of ${{\text {H}}^{\text {N}}}$, N, ${{\text {C}}_\alpha }$, and C$^{\prime }$ is more beneficial than adding ${{\text {C}}_\beta }$ CSs. Our program for LDA analysis is available at https://github.com/gugumatz/LDA-Temp-Coeff.

**Supplementary Information:**

The online version contains supplementary material available at 10.1007/s10858-024-00452-9.

## Introduction

Intrinsically disordered proteins (IDPs) are widespread and play essential biological roles, especially in molecular recognition, signaling, and regulatory processes (Dunker et al. 2001; Dyson and Wright 2005). Most IDPs are impossible to crystalize; thus, liquid-state NMR remains the key technique to study their nature. The NMR analysis usually starts with the resonance assignment. Typically, we establish sequential connectivities of chemical shifts and then form spin-system chains that are eventually mapped onto the protein sequence. The mapping can be executed by recognizing residues corresponding to characteristic amino-acid types. Due to the low dispersion of chemical shifts in IDPs (especially ${\text {H}}^{{\text {N}}}$), it is often beneficial to employ high-dimensional techniques, which better resolve spectral peaks and thus reduce the number of ambiguities during the chains’ formation (Kazimierczuk et al. 2013; Brutscher et al. 2015; Grudziaz et al. 2018). Also, using $^{13}{\text {C}}$-detected experiments (Felli and Pierattelli 2022) can increase the chains’ lengths, as the presence of proline residues does not break the chain, as it is when detecting the amide proton signal. Another possibility to facilitate chains mapping is based on amino-acid selective experiments (Dötsch et al. 1996), later extensively developed (Schubert et al. 2005; Pantoja-Uceda and Santoro 2008; Piai et al. 2016). Such an approach requires recording several experiments, each providing signals of a different group of amino-acid residues. More widely used are the methods based on chemical shift statistics for different residues [e.g. using BioMagResBank (Ulrich et al. 2007)]. However, manual analysis of such multidimensional data (chemical shifts of several nuclei types) is difficult. One of the tools for automating the analysis process and assigning ambiguous amino acid residues is advanced statistical method, e.g., linear discriminant analysis (LDA), which can automatically recognize the type of amino acid residue (Romero et al. 2022).

Once the resonance assignment is done, NMR can be used to study the structure and dynamics of a protein. For example, tracking changes in NMR chemical shifts with temperature is a sensitive indicator of solvent exposure (Stevens et al. 1980). In typical temperature ranges, the changes are linear and are described with a temperature coefficient (TC), corresponding to a slope of the dependence. The deviations from linearity indicate the presence of low-populated excited states (Doyle et al. 2016; Cierpicki et al. 2002). TCs (different for each nucleus) are typically measured using a series of spectra acquired at different temperatures. Alternative methods such as non-stationary NMR (Shchukina et al. 2021) or techniques based on non-uniform sampling (Bermel et al. 2014; Shchukina et al. 2023) have been also proposed.

**Fig. 1:**
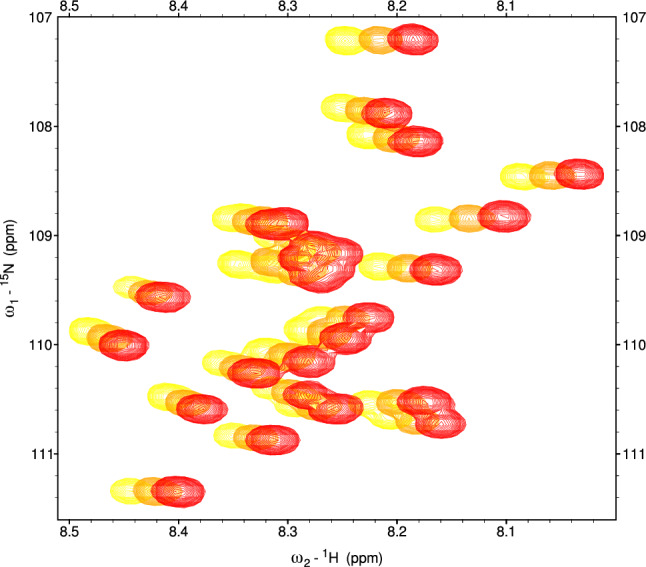
A glycine region of stacked $^{15}{\text {N}}$-HSQC spectra of the studied 1-239 Tau protein fragment acquired at different temperatures: $5^\circ$C (red), $10^\circ$C (orange) and $15^\circ$C (yellow). It can be seen that peaks corresponding to different glycine residues shift at different rates

It has been reported in the past (Baxter and Williamson 1997), that protein temperature coefficients, similarly to chemical shifts, are also residue-specific. Thus, they seem to be a promising parameter that may facilitate amino-acid type recognition and support resonance assignment. Yet, TCs of different residues of the same amino-acid type are not identical (see Fig. 1). The previous studies (Baxter and Williamson 1997; Cierpicki and Otlewski 2001) showed that temperature coefficient values largely depend on the local structure. However, they are difficult to predict theoretically (Baxter and Williamson 1997). Even random coil chemical shift calculators that take temperature into account (Kjaergaard et al. 2011; Nielsen and Mulder 2018) apply relevant correction in a simplified way, i.e., the same for all residues of a given type.

This paper exploits TCs to support resonance assignment using a data-driven approach instead of theoretical predictions. We propose using TCs as extra dimensions in the aforementioned linear discriminant analysis, in addition to chemical shifts. Since no widely available databases contain TCs for IDPs, we construct a training data set using easily assignable protein fragments under investigation. We validate the proposed approach using an intrinsically disordered Tau protein construct (residues 1-239) (Ukmar-Godec et al. 2020).

## Methods

### Protein expression and purification

To produce the U-$^{15}{\text {N}}$, $^{13}{\text {C}}$ Tau239 protein, the vector pNG2 (a derivative of pET-3a, Merck-Novagen, Darmstadt) codifying for its gene was transformed into E. coli BL21(DE3) competent cells. These were grown using Luria-Bertani (LB) medium supplemented with Ampicillin at $37^\circ$C until they reached an $\hbox {OD}_{{600}}$ of 0.6 to 0.8. At this point, the cells were centrifuged at low speed, washed with M9 salts (${{\text {Na}}_2{\text {HPO}}_4}$, ${{\text {KH}}_2{\text {PO}}_4}$, and NaCl), centrifuged again and resuspended in $\frac{1}{4}$ of the initial LB volume M9 minimal medium containing ${ ^{15}{\text {NH}}_4{\text {Cl}}}$ (1 g/L) and U-${^{13}}$C${_{6}}$-D-glucose (2 g/L) as the only nitrogen and carbon source respectively. Then, the cell culture was incubated for 1 hr at $37^\circ$C and induced with 1.0 mM isopropyl ${\beta }$-D-1-thiogalactopyranoside (IPTG) overnight. Next, the cells were harvested through centrifugation and the resulting pellet was resuspended using a lysis buffer 20 mM MES pH 6.8, 1 mM EGTA, 2.0 mM dithiothreitol (DTT), protease inhibitor cocktail, 1.0 mM ${{\text {Mg}}}{\text {Cl}}_{2}$, deoxyribonuclease (DNase) I and lysozyme. After incubation, a French press was used to disrupt the cells. Then, sodium chloride (NaCl) was added to reach a final concentration of 500 mM, followed by a 20 min boiling step. To remove all denatured proteins, the sample was ultracentrifuged at 127,000$\times$g and $4^\circ$C. The DNA in the solution was precipitated by adding 20 mg/mL of streptomycin sulfate to the supernatant. Then, after incubation for 15 min, the sample was centrifuged at 15,000$\times$g, and the DNA pellet was removed. The precipitation of Tau239 was induced by adding 0.361 g/mL ammonium sulfate ${({\text {NH}}_4)_2{\text {SO}}_4}$ followed by the 15 min incubation and centrifugation at 15,000$\times$g. The resulting pellet of Tau239 was resuspended and dialyzed against buffer 20 mM MES pH 6.8, 1.0 mM EDTA, 2.0 mM DTT, 0.1 mM phenylmethylsulfonyl fluoride (PMSF) at $4^\circ$C overnight. The sample recovered from the dialysis was then loaded onto an ion-exchange chromatography column where the Tau239 was purified from its contaminants. Finally, Tau239 protein was dialyzed against 50 mM sodium phosphate (NaP), pH 6.8, and concentrated by ultrafiltration (3 KDa Viva spin from Sartorius).

### NMR spectroscopy

The NMR sample was prepared with 100 ${\mu }$M U-$^{15}{\text {N}}$, $^{13}{\text {C}}$ Tau239 in 50 mM sodium phosphate buffer pH 6.8 and 10% ${{\text {D}}_2{\text {O}}}$. The NMR experiments were performed on a Bruker 800 MHz spectrometer equipped with a triple-resonance cryogenic probe and Avance NEO console. The following triple-resonance experiments were recorded using the Bruker pulse sequence library: HNCO (Kay et al. 1990), HN(CA)CO (Yang and Kay 1999), HNCA (Kay et al. 1990), HN(CO)CA (Sattler et al. 1999), and (HAHB)CACB(CO)NH. The complete set of NMR experiments was collected at $5^\circ$C. The experiments HNCO, HN(CO)CA, and (HAHB)CACB(CO)NH (Muhandiram and Kay 1994) were collected for $10^\circ$C and $15^\circ$C. All NMR experiments were acquired using non-uniform sampling and processed using a CS module of MDDNMR program (iterative soft thresholding algorithm, 200 iterations, virtual echo option) (Orekhov et al. 2004-2024; Mayzel et al. 2014). The experimental parameters are shown in Table 1. All spectra were displayed and analyzed using the Sparky program (Lee et al. 2015).

The theoretical chemical shift vs. temperature dependence is not linear, but sigmoidal. Moreover, the deviations from linearity coherent for all peaks may result from inaccurate referencing and other instrumental factors. In any case, for LDA it is better to abandon the traditional determination of TC as the global slope. Instead, we determined separately $\hbox {TC}_{5-10}$, reflecting changes between 5 and $10^\circ$C, and $\hbox {TC}_{10-15}$, reflecting changes between 10 and $15^\circ$C.

**Table 1:** NMR acquisition parameters

Experiment	$t_{max}$ for $^{13}{\text {C}}$ dim. (ms)	NS	NUS points (% sampling)
HNCO_sct	50.54	4	600 (7.03%)
HN(CA)CO_sct	50.54	16	1000 (11.70%)
HNCA_sct	20.10	8	600 (4.60%)
HN(CO)CA_sct	20.10	8	450 (3.45%)
(HBHA)CBCA(CO)NH	66.29	8	1000 (8.92%)

The maximum evolution times in ${{\text {N}}^{\text {H}}}$ and ${\text {H}}^{{\text {N}}}$ dimensions in all experiments were set to 71.96 ms and 71.68 ms, respectively. The maximum evolution times for $^{13}{\text {C}}$ dimension are listed in the second column. The **NS** stands for the number of scans. The acronym ‘sct’ stands for semi-constant time

## Results and discussion

Linear discriminant analysis is a classification method that calculates the probability that a multidimensional data point belongs to one of the user-defined classes. The algorithm, trained with data points belonging to known classes, finds a combination of dimensions that minimizes the variance within each class and maximizes the variance between classes (Balakrishnama and Ganapathiraju 1998; Tharwat et al. 2017). In our previous work (Romero et al. 2022), we used LDA to find the most differentiating combination of chemical shifts, allowing us to assign a new spin system to one of the amino acid residue types. In this study, we undertake a similar task using not only chemical shifts, but also TCs.

Previously, we used chemical shifts of IDPs deposited in BMRB (Romero et al. 2022) for the training step. However, such an approach would not be feasible for TCs since a relevant database does not exist. A possible solution is to use partial resonance assignment of the studied protein to train the algorithm, which will be later used to classify the not-assigned parts. There are many practical situations in which such a partial assignment is available. One of them is when we want to transfer the resonance assignment performed on a different sample of the same protein (e.g., measured under slightly different conditions). In such a case, some peaks are often well-separated and can be unambiguously assigned, while others are in crowded regions or severely shifted with respect to the original peak list. Another situation is that in the course of sequential assignment, some chains can be easily mapped on the protein sequence, but others are not sufficiently long or characteristic. Mapping the latter chains requires sophisticated methods of residue-type recognition, like LDA, that can also exploit the “easy part” of the data for training. Of course, an approach exploiting data from the same protein for training is justified only for relatively large proteins—for others, the number of assigned residues of each amino acid type can be too small to train the algorithm effectively. Also, the chemical shift values have to be dependent predominantly on the residue type, thus the approach applies to proteins of disordered nature. We demonstrate the method using spin systems from a Tau protein fragment of 239 residues containing 28 glycines and 26 prolines. Peaks corresponding to 141 residues from the peak list previously deposited in BMRB (entry 28065) fitted well to our spectra acquired at $5^\circ$C and were used for training. As discussed below, the ambiguities in assigning the 19 spin systems have been solved using LDA. For training and testing, we considered only residues for which complete sets of chemical shifts (${\text {H}}^{{\text {N}}}$, N, C$^{\prime }$, ${\text {C}}_{\alpha }$, ${\text {C}}_{\beta }$) could be clearly found from our spectra. The glycines were excluded from the analysis since their assignment was, as usual, rather obvious. The remaining 22 resonances (not counting the N-terminal one) were missing.

**Fig. 2:**
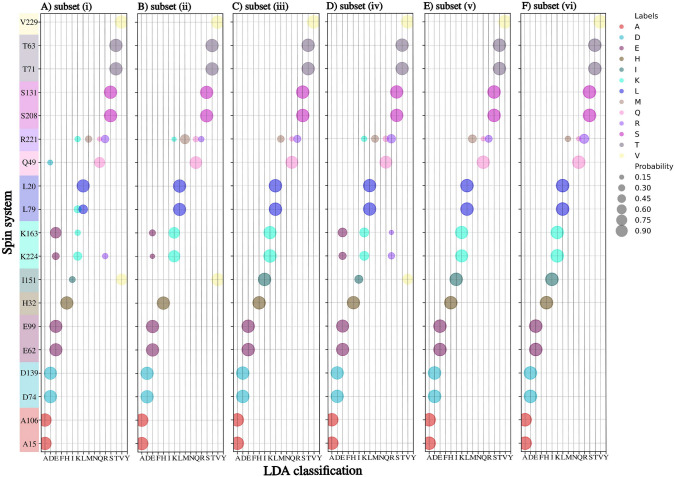
Results of linear discriminant analysis of CSs and TCs of 19 Tau 1-239 residues. The residues for which peak list transfer (from BMRB entry 28065) was ambiguous are presented. **A** subset (i): ${\text {H}}^{{\text {N}}}$, N, C', ${\text {C}}_{\alpha }$; **B** subset (ii): ${\text {H}}^{{\text {N}}}$, N, C', ${\text {C}}_{\alpha }$ and their $\hbox {TC}_{5-10}$; **C** subset (iii): ${\text {H}}^{{\text {N}}}$, N, C', ${\text {C}}_{\alpha }$ and their $\hbox {TC}_{5-10}$ and $\hbox {TC}_{10-15}$; **D** subset (iv): ${\text {H}}^{{\text {N}}}$, N, C', ${\text {C}}_{\alpha }$ and ${\text {C}}_{\beta }$; **E** subset (v): ${\text {H}}^{{\text {N}}}$, N, C', ${\text {C}}_{\alpha }$, ${\text {C}}_{\beta }$ and their $\hbox {TC}_{5-10}$; **F** subset (vi): ${\text {H}}^{{\text {N}}}$, N, C', ${\text {C}}_{\alpha }$, ${\text {C}}_{\beta }$, and their $\hbox {TC}_{5-10}$ and $\hbox {TC}_{10-15}$

Figure 2 shows the results of using LDA on a 1-239 Tau fragment with and without TCs. We constructed six sets of CSs and TCs of different nuclei: subset (i) ${\text {H}}^{{\text {N}}}$, N, C$^{\prime }$, ${\text {C}}_{\alpha }$ CSs; subset (ii): ${\text {H}}^{{\text {N}}}$, N, C$^{\prime }$, ${\text {C}}_{\alpha }$ CSs and $\hbox {TC}_{5-10}$; subset (iii): ${\text {H}}^{{\text {N}}}$, N, C$^{\prime }$, ${\text {C}}_{\alpha }$ CSs and $\hbox {TC}_{5-10}$ and $\hbox {TC}_{10-15}$; subset (iv): ${\text {H}}^{{\text {N}}}$, N, C$^{\prime }$, ${\text {C}}_{\alpha }$ and ${\text {C}}_{\beta }$ CSs; subset (v): ${\text {H}}^{{\text {N}}}$, N, C$^{\prime }$, ${\text {C}}_{\alpha }$, ${\text {C}}_{\beta }$ CSs and $\hbox {TC}_{5-10}$; subset (vi): ${\text {H}}^{{\text {N}}}$, N, C$^{\prime }$, ${\text {C}}_{\alpha }$, ${\text {C}}_{\beta }$ CSs and $\hbox {TC}_{5-10}$ and $\hbox {TC}_{10-15}$.

Adding $\hbox {TC}_{5-10}$ to a set of ${\text {H}}^{{\text {N}}}$, N, C$^{\prime }$, and ${\text {C}}_{\alpha }$ CSs (Fig. 2B) allows unambiguously recognizing lysine, leucine, and glutamine residues, which were not recognized by CSs only. Although, in one case, adding $\hbox {TC}_{5-10}$ causes misclassification of isoleucine residue (I151 is recognized as valine), the problem is solved by adding the $\hbox {TC}_{10-15}$. We get better results in subset (iii) than in subset (iv). Thus, when ${\text {C}}_{\beta }$ CS is not available, good variable-temperature data (e.g., for three different temperatures) can replace it. The most efficient is a subset (vi) (Fig. 2F). Generally, it correctly classifies amino acid residues, except for arginine, which is assigned to three classes. Nonetheless, the arginine has the highest probability (above 50%) of these three. As can be seen, the subset (vi) is only slightly better than (v) but requires collecting more data at higher temperatures, which is time-consuming and may be problematic in the case of not stable protein. Thus, we will use subset (v) for further examples discussed below. It is not crucial at which temperatures the spectra are acquired as long as differences in chemical shifts are residue type-specific, the protein is stable and amide proton chemical exchange does not hamper the measurement.

Notably, even when only CSs are used, training based on different parts of the Tau protein is optimal and increases classification efficiency compared to BMRB-based training discussed in our previous work (Romero et al. 2022) (see Supplementary Information Fig. S1). This might be caused by the temperature used in our experiment ($5^\circ$C) being very different from the typical temperatures in the BMRB entries from the training set.

Another example application of LDA, besides peak list transfer, is mapping spin-system chains formed during sequential assignment on the protein sequence. The process is generally easier for long chains containing residues with characteristic chemical shifts (i.e., alanines, glycines, serines, and threonines). However, the chains are often interrupted when peaks are missing due to fast nuclear relaxation, chemical exchange, peak overlap, or lack of ${\text {H}}^{{\text {N}}}$ at proline residues. Unambiguous mapping of such short chains in a large protein is often difficult.

**Fig. 3:**
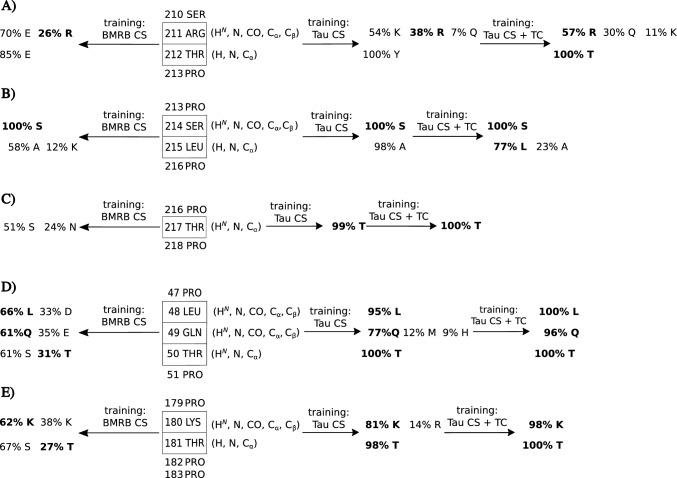
Comparison of LDA-based amino acid type recognition in short chains using 3 training data sets: BMRB, chemical shifts from the same protein (Tau 1-239) with and without TCs. LDA was performed using subset (iv): ${\text {H}}^{{\text {N}}}$, N, C', ${\text {C}}_{\alpha }$, ${\text {C}}_{\beta }$; and subset (v): ${\text {H}}^{{\text {N}}}$, N, C', ${\text {C}}_{\alpha }$, ${\text {C}}_{\beta }$, and their TCs. The recognitions with probability scores exceeding 10% are shown, and the correct residue type is marked in bold. Panels **A**–**E** show the recognition of different short chains

In the studied Tau fragment, several short chains between prolines were present. Figure 3 compares the efficacy of amino acid type recognition in these chains using LDA with three different kinds of training data: CSs from BMRB, and CSs from the same protein (Tau 1-239) with and without $\hbox {TC}_{5-10}$. We used the same training data for the latter two as for Fig. 2. Some of the short chains could not be mapped using LDA with CS-only BMRB-based training (Fig. 3, left side). In contrast, by training using data from the same protein in three of the shown cases (Fig. 3, panels C, D, E) the chains could be correctly mapped. For chains shown in Fig. 3a, b, the ambiguity still remains but is resolved by $\hbox {TC}_{5-10}$.

Let us discuss the short-chain identification from Fig. 3 in more detail. For the chain shown in panel A), LDA trained with chemical shift data from BMRB wrongly classifies the 211Arg, although a complete set of chemical shifts for this residue is available (${\text {H}}^{{\text {N}}}$, N, C', ${\text {C}}_{\alpha }$ and ${\text {C}}_{\beta }$). The correct classification has the second highest probability (26%). Using training data from the same protein increases it by 10%, but still, the classification is wrong. The additional use of $\hbox {TC}_{5-10}$ resulted in the correct amino acid recognition (at the level of 57%). Another residue in the same chain—212Thr—is also misclassified if CS-only data is used (although only ${\text {H}}^{{\text {N}}}$, N and ${\text {C}}_{\alpha }$), but with $\hbox {TC}_{5-10}$, the 100% correct classification is achieved). A similar scenario is repeated for the 215Leu from Fig. 3b). Examples shown in Fig. 3c–e) present the superiority of the “same protein” approach over BMRB-based training. For all residues, the correct classification is better with the former approach. 217Thr, 50Thr, and 181Thr are properly classified only using LDA trained on chemical shifts from the same protein. Importantly, these are the spin systems with incomplete sets of chemical shifts (only ${\text {H}}^{{\text {N}}}$, N and ${\text {C}}_{\alpha }$). The additional use of $\hbox {TC}_{5-10}$ improves the correct classification even more.

## Conclusions

In this paper, we showed that temperature coefficients can be used to support the resonance assignment of intrinsically disordered proteins. A similar approach, however without LDA, has recently been applied to support resonance assignment in small molecules (Nawrocka et al. 2024). Although the relationship between amino acid residue type and TC values is neither strict nor straightforward, the linear discriminant analysis can find it. We believe that creating temperature coefficient databases would enable wider use of approaches like the one described.

## Supplementary information

The linear discriminant analysis of chemical shifts of Tau protein fragment (1-239) using two approaches based solely on chemical shifts (not TCs): training data generated from BMRB (proteins other than Tau) vs. training data from the assigned part of the same protein.

## Supplementary Information

Below is the link to the electronic supplementary material.

Supplementary file 1 (pdf 1065 KB)

## Data Availability

The experimental data—raw signals, NUS processing scripts and Sparky files—are available at zenodo.org (10.5281/zenodo.11500505). The LDA program is available at https://github.com/gugumatz/LDA-Temp-Coeff.
