# Supplementary material for: Using temperature coefficients to support resonance assignment of intrinsically disordered proteins
Source: J Biomol NMR. 2024 Dec 7;79(1):59–65. doi: 10.1007/s10858-024-00452-9 (PMC11832634; doi:10.1007/s10858-024-00452-9)
Supplement: Supplementary file 1 — (pdf 1065 KB) [file 10858_2024_452_MOESM1_ESM.pdf]

Supplementary Information to ”Using temperature coefficients to support resonance assignment of intrinsically disordered proteins”

Paulina Putko[1], Javier A. Romero[1], Christian F. Pantoja[3], Markus Zweckstetter[3], Anna Zawadzka-Kazimierczuk[†2], Krzysztof Kazimierczuk[*1]

[1] *Centre of New Technologies, University of Warsaw, Banacha 2C, 02-097 Warsaw, Poland*
[*] *k.kazimierczuk@cent.uw.edu.pl* .
[2] *Biological and Chemical Research Centre, Faculty of Chemistry, University of Warsaw, Żwirki i Wigury 101, 02-089 Warsaw, Poland*
[†] *anzaw@chem.uw.edu.pl.*
[3] *German Center for Neurodegenerative Diseases (DZNE), 37075, Göttingen, Germany Department for NMR-based Structural Biology, Max Planck Institute for Multidisciplinary Sciences, 37077, Göttingen, Germany.*

Our previous research (Romero et al., 2022) has shown that advanced statistical methods, like linear discriminant analysis, can support the assignment of spin systems to the residue types in IDPs. Training LDA on IDP assignments from the BMRB database helps recognize the type of amino acid residues. However, as shown in Fig. S1, using the same protein for both training and testing provides better results since such an approach is not vulnerable to differences in measurement conditions. It is possible to correctly classify the following AAs in each test set: alanine, aspartic acid, serine, threonine, valine, and tyrosine.

When the number of chemical shifts is limited to H^N, N, C', and C_α (see Figure S1A and S1B), training and testing on one protein can distinguish among similar amino acid types: glutamine and glutamic acid, and also recognize arginine with higher

probability. Additional use C_β improved recognition of leucine (Fig. S1 C). Admittedly, there are exceptions - using the BMRB database enabled better classification of isoleucine and lysine (Fig. S1 D).

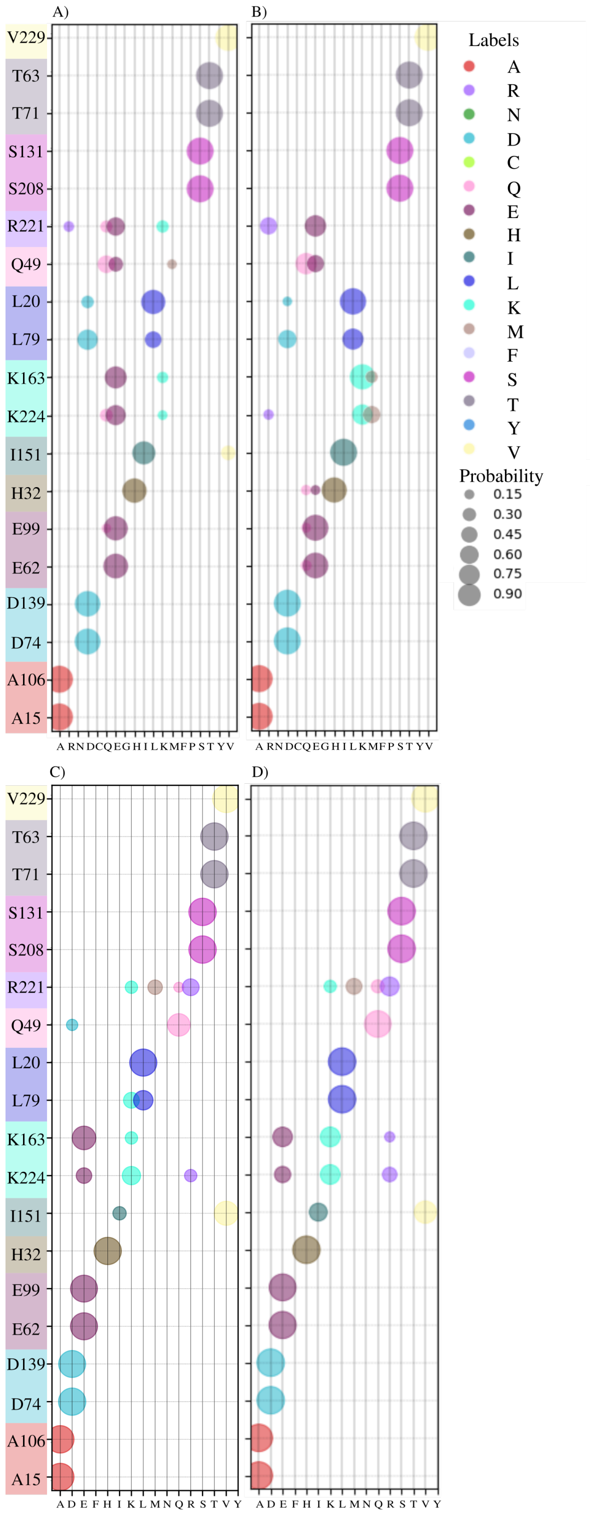

Fig. S1 Results of linear discriminant analysis of the chemical shifts. Panels A) and C) present the results from LDA trained on the assigned part of the studied protein. Panels B) and D) present the results from LDA trained on BMRB data. Panels A) and B) show results from chemical shifts of H^N, N, C', and C_α, panels C) and D) from H^N, N, C', C_α and C_β of 19 spin systems of Tau 1-239.

BMRB entries used for training

17836, 18767, 25044, 25426, 26530, 26740, 27426, 27553, 27554, 30773, 50496, 50497, 51116, 51147, 51172, 51286, 51353, 51354, 51355, 4922, 17482, 26544, 26554, 26841, 27351, 28121, 50803, 50944, 51420, 15719, 51011, 19246, 18417, 25327, 26770, 27179, 50961, 25921, 6968, 11526, 16296, 17290, 15176, 15179, 6436, 15180, 16445, 16450, 17483, 19999, 25118, 15225, 15430, 51301

References

Romero, J.A., Putko, P., Urbańczyk, M., Kazimierczuk, K., Zawadzka-Kazimierczuk, A.: Linear discriminant analysis reveals hidden patterns in NMR chemical shifts of intrinsically disordered proteins. PLoS Computational Biology **18**, 1010258 (2022)
